# RgnTX: Colocalization analysis of transcriptome elements in the presence of isoform heterogeneity and ambiguity

**DOI:** 10.1016/j.csbj.2023.08.021

**Published:** 2023-08-24

**Authors:** Yue Wang, Zhen Wei, Jionglong Su, Frans Coenen, Jia Meng

**Affiliations:** a Department of Mathematical Sciences, Xi’an Jiaotong-Liverpool University, Suzhou, Jiangsu 215123, China; b Department of Biological Sciences, Xi’an Jiaotong-Liverpool University, Suzhou, Jiangsu 215123, China; c School of AI and Advanced Computing, Xi’an Jiaotong-Liverpool University, Suzhou, Jiangsu 215123, China; d AI University Research Centre, Xi’an Jiaotong-Liverpool University, Suzhou, Jiangsu 215123, China; e Department of Computer Science, University of Liverpool, L69 7ZB Liverpool, United Kingdom; f Institute of Systems, Molecular and Integrative Biology, University of Liverpool, L69 7ZB Liverpool, United Kingdom

**Keywords:** Permutation test, Isoform ambiguity, Colocalization analysis, Multiple hypothesis testing, RNA methylations, Transcriptome

## Abstract

Colocalization analysis of genomic region sets has been widely adopted to unveil potential functional interactions between corresponding biological attributes, which often serves as the basis for further investigation. A number of methods have been developed for colocalization analysis of genomic elements. However, none of them explicitly considered the transcriptome heterogeneity and isoform ambiguity, making them less appropriate for analyzing transcriptome elements. Here, we developed RgnTX, an R/Bioconductor tool for the colocalization analysis of transcriptome elements with permutation tests. Different from existing approaches, RgnTX directly takes advantage of transcriptome annotation, and offers high flexibility in the null model to simulate realistic transcriptome-wide background, such as the complex alternative splicing patterns. Importantly, it supports the testing of transcriptome elements without clear isoform association, which is often the real scenario due to technical limitations. Proposed package offers a wide selection of pre-defined functions, easy to be utilized by users for visualizing permutation results, calculating shifted z-scores and conducting multiple hypothesis testing under Benjamini-Hochberg correction. Moreover, with synthetic and real datasets, we show that RgnTX novel testing modes return distinct and more significant results compared to existing genome-based methods. We believe RgnTX should make a useful tool to characterize the randomness of the transcriptome, and for conducting statistical association analysis for genomic region sets within the heterogeneous transcriptome. The package now has been accepted by Bioconductor and is freely available at: https://bioconductor.org/packages/RgnTX**.**

## Graphical Abstract

**Figure:**
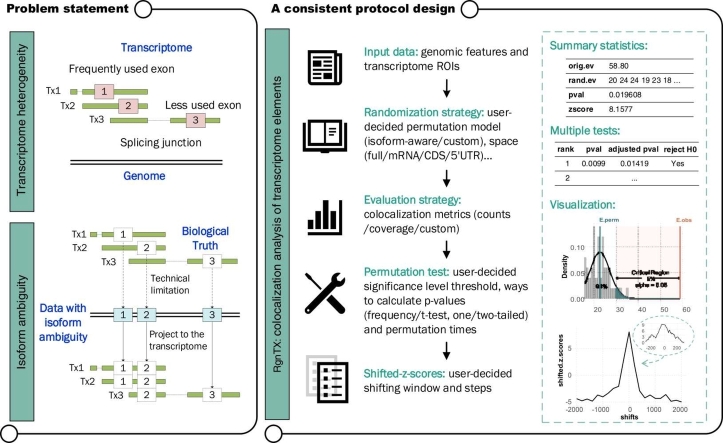

## 1 Introduction

One of the primary outputs of many high-throughput methods is the genomic regions [1], [2]. Genomic regions, associated with specific biological features, such as open chromatin regions, protein binding sites, and microRNA target sites, are routinely produced from various biotechnologies and analysis pipelines, e.g., the transcription factor binding sites identified from ChIP-seq technique with MACS software [3] and the RNA binding protein target sites predicted computationally [4]. Such genomic region sets have also been commonly used to functionally annotate the genome and spatially characterize relevant biological attributes [5]. Integrative analyses of genomic attributes have revealed that such characteristics are nonrandomly distributed in the genome, with the assumption that corresponding enrichment might be biologically relevant [6], [7].

To infer potential interactions between biological attributes, colocalization analysis (also termed as ‘co-occurrence’ or ‘spatial correlation analysis’) of corresponding genomic region sets has been widely used. This analysis summarizes the pairwise relationship between region sets as a test statistic, which is then subjected to statistical testing to determine whether the observed result is likely to have occurred by chance [2], [8]. Computational tools and suitable strategies to perform colocalization analysis among genomic regions are needed to increase our understanding of potential functional interactions and provide hints for further investigation. For example, colocalization of the binding sites of two transcriptional factors may suggest their synergetic action [9]; the spatial correlation of histone modification and RNA modification unveiled the guiding role of H3K36me3 on m6A methylation in a co-transcriptional manner [10]. To be noticed, colocalization analysis discussed here is different from the Genome-Wide Association Studies (GWAS) or expression Quantitative Trait Loci (eQTL) studies. GWAS and eQTL focus on studying association between single nucleotide polymorphisms (SNPs) with diseases, traits or gene expression changes, while the colocalization analysis in this research aims to explore the spatial association between specific genomic region sets.

The null models for colocalization analysis can be constructed using either analytical tests or permutation tests (Monte Carlo simulations) [2]. Analytical tests make inferences about population parameters based on assumptions about the underlying data distribution, such as GREAT (binomial test) [11], LOLA (Fisher’s exact test) [12], GenomeRunner (chi-square test and binomial test) [13] and nearBynding (Welch’s t-test) [14]. On the other hand, permutation tests, i.e., methods incorporate Monte Carlo simulations, create a null distribution of the test statistic by repeatedly sampling data, such as BEDTools [15], HyperBrowser [16], MULTOVL [17], IntervalStats [18], GenometriCorr [19], GAT [20], BITS [21], ChIPseeker [22], regioneR [23], StereoGene [24], OLOGRAM [25] and Bedshift [26]. Compared to purely analytical methods, permutation tests offer greater flexibility in selecting appropriate test statistics and null models, but they require more computational resources. Furthermore, these methods have led to the development of functional annotation web servers for genomic regions, such as GIGGLE [27], LOLAweb [28] and Coloc-stats [8], which have greatly facilitated the analysis of genomic region datasets. A review article provides an overview of current tools for colocalization analysis and presents a summary of these methods in tabular form [29].

**Fig. 1:**
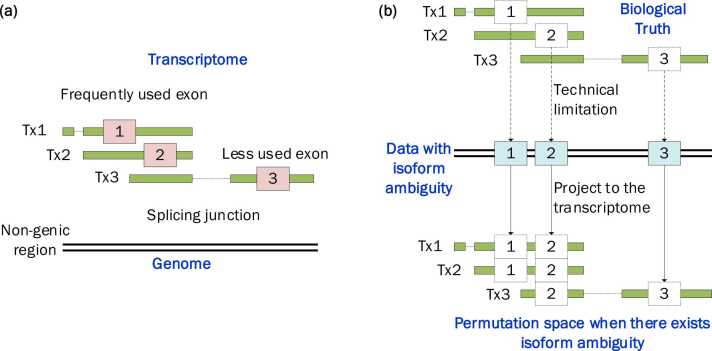
Transcriptome heterogeneity and isoform ambiguity problem in colocalization analysis. **a)** Compared with the linear genome space, transcriptome space is heterogeneous due to the existence of multiple isoform transcripts and the splicing junctions. **b)** The isoform specificity of transcriptome elements is often lost in the process of mapping due to technical limitations. It is unclear which specific isoform transcript is associated with a transcriptome element. Genome-based methods that shuffle features over the genome are not capable of analyzing genomic elements with the transcriptome heterogeneity and the isoform ambiguity problem.

It is a non-trivial task to make an informed choice about the null models according to the distributional properties and dependence structure of the genomic features [2]. However, existing approaches have a fundamental limitation as they were primarily designed for genome-based analysis and do not account for the heterogeneity of the transcriptome (Fig. 1**a**), i.e., multiple independent isoform transcripts in formality, but with shared exons when mapped (or projected) to the genome. The genome colocalization methods does not consider RNA structures and is therefore not suitable for analyzing transcriptome regions, such as 3′UTRs or RNA methylation sites. Another widely existing problem is the isoform ambiguity (Fig. 1**b**), where it is unclear which specific isoform transcript is associated with a transcriptome element, because it overlaps with multiple isoforms when mapped to the genome, which is often the real scenario in biological research. Although long-read technologies like Nanopore direct RNA sequencing enable isoform-specific identification of biological attributes [30], most existing technologies and databases still could not provide isoform-specificity. For example, the m^6^A RNA methylation sites reported from MeRIP-seq [31] and the RNA-binding protein target sites collected in POSTAR3 database [32] lack information about the specific isoform they originate from. To date, there are no computational methods available to address the issue of isoform ambiguity during transcriptome element colocalization analysis.

Here we developed RgnTX, an R/Bioconductor tool for the colocalization analysis of transcriptome elements with permutation tests. The main permutation test framework is inspired by the regioneR [23]. Different from existing approaches, RgnTX offers high flexibility in the null model to simulate realistic transcriptome-wide background. It allows user-decided restriction of areas at transcriptome level during the shuffling, which provides control over shuffling related to the type (pre-mRNA, mRNA, 5′UTR, CDS and 3′UTR) and the combination of any specific transcripts. Importantly, RgnTX is able to cope with the isoform ambiguity problem in colocalization analysis. Although it does not provide a solution for whether a genomic feature with isoform ambiguity is overlapped with a specific transcriptome region, it is capable of calculating a weighted overlapping count as metrics to measure the association between such features and some category of transcriptome regions, for example the association between m^6^A RNA methylations and stop codons, where the m^6^A sites derived from the RNA-seq usually have isoform ambiguity issue. Besides, RgnTX supports conducting multiple hypothesis testing with Benjamini-Hochberg correction. It reports the proportion of hypothesis tests with the null hypothesis being rejected among all tests based on a user-specified significance threshold.

By utilizing randomly selected data, we revealed that the null distribution of random overlaps generated by the permutation model over the genome and the transcriptome could be significantly different. With the tests on m^6^A data, we showed that the novel testing modes provided by RgnTX return distinct and more significant results than the existing genome-based methods. RgnTX should make a potentially powerful tool for characterizing the randomness of transcriptome and performing statistical association analysis on genomic region sets within the heterogeneous transcriptome.

## 2 Material and methods

### 2.1 Randomization strategies and null models

RgnTX is written in R and utilizes the IRanges, GenomicRanges and GenomicFeatures structures [33]. The simplest randomization strategy supported by RgnTX is to shuffle each feature over the whole transcriptome without any restrictions. To perform this randomization, users only need to provide the desired number of regions to be selected, along with the region length, and specify a UCSC txdb object (default is hg19) to the randomization function with a single line of code. The output of this process is a GRangesList object representing the randomly picked region set, which includes information of chromosome names, start/end positions of each interval, strand types, and transcript IDs associated with each randomized region.

Multiple randomized region sets can also be generated with just one line of code.

Nevertheless, it is often overly simplistic to permutate regions over the whole transcriptome, as this can result in an overestimation of colocalization in region set association analysis. Here, RgnTX provides a method for performing isoform-aware permutation for transcriptome elements. In this method, each feature is first projected to the genome and then only permutated over the transcripts that overlap with it. Fig. S1 provides an illustration of this process. Feature 3 and 4 are only permutated within transcript Tx3, while feature 2 is permutated within all three transcripts. The random regions are generated from the same transcript (or potential transcripts) as the original RNA features, thereby preserving the characteristics of features and controlling potential confounding factors. This randomization strategy is used as the default null model in the colocalization analysis protocol, so as to preserve the local heterogeneity of each feature and retain maximal isoform information.

**Fig. 2:**
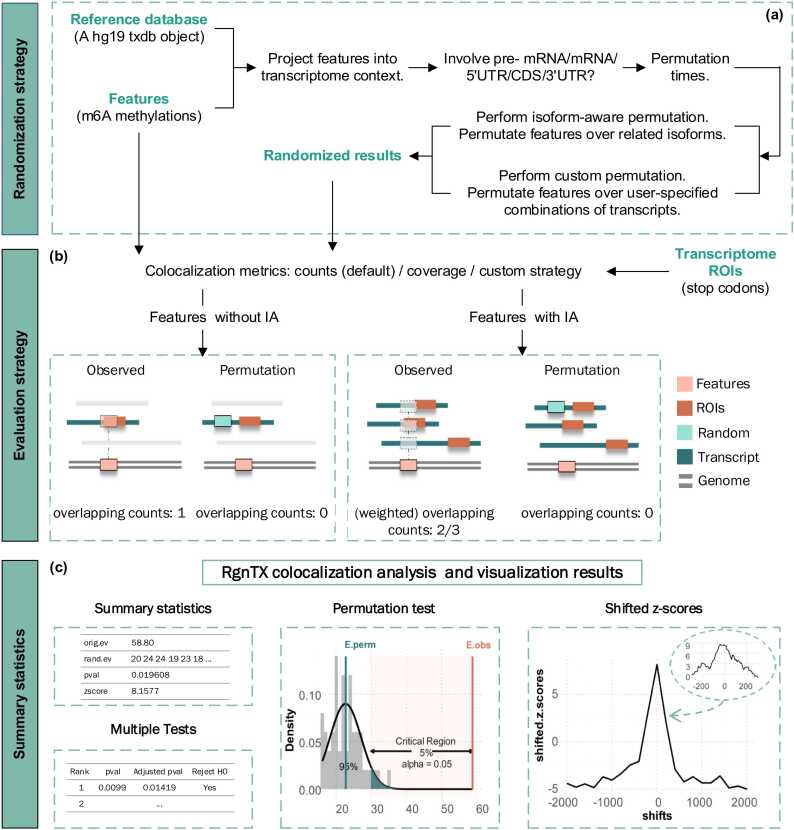
RgnTX workflow and results. **a)** Randomization strategy. RgnTX supports permutation over the entire transcriptome, over the set of relative isoforms of each feature (isoform-aware permutation) and over any user-specified combination of transcripts (custom permutation) with freedom choice of including introns or not with any permutation times. **b)** Evaluation strategy. RgnTX provides separate evaluation strategy for features with and without the isoform ambiguity (IA) problem, making observed estimate and random estimates to be compared based on the same scale. **c)** Results obtained by RgnTX. A consistent protocol design is implemented to make the package easy to use. Summary statistics generated by the core permutation test function are taken as the input of the plotting functions, which generate two types of graphical outputs.

Moreover, RgnTX allows for the randomization within specific types of the transcriptome background such as pre-mRNA, mRNA(exons), 5′UTR, CDS and 3′UTR via corresponding argument in randomization functions. RgnTX also supports shuffling features across any combination of transcripts. To sum, it supports permutation over the entire transcriptome, over relative isoforms of each feature, and over any user-specified combination of transcripts, with the option to include or exclude introns. This flexibility provides the ability to simulate a realistic transcriptome-wide background based on the user's requirements (Fig. 2**a)**. For more detailed information about the RgnTX package, please refer to the package vignettes on its Bioconductor landing page.

### 2.2 Evaluation strategies

The evaluation strategy aims to define a test statistic summarizing the relation between two region sets. In RgnTX, the default evaluation strategy is counting the overlapping regions at the transcriptome level. Unlike traditional methods that detect overlapping based on the genome, in this strategy, two regions that have shared intervals on the genome but are located on different transcripts are considered not to have overlap with each other.

Another important factor to consider is isoform ambiguity (IA). To date, there are no computational methods to cope with isoform ambiguity in transcriptome-wide colocalization analysis. To tackle this ambiguity problem, here a novel evaluation strategy has been devised (Fig. 2**b**). A weighted overlapping count is calculated as a measure of colocalization between *S* features (with IA) and a specific category of transcriptome regions (without IA), what we call transcriptome regions of interest (ROIs):

$$C_{\mathrm{weigh}}:=\sum_{i=1}^{S}c^{\left(i\right)}/n^{\left(i\right)}$$

where each feature, denoted as the *i* -th feature, $n^{\left(i\right)}$ is the total count of related isoforms of the feature, while $c^{\left(i\right)}$ is the count that the feature is overlapped with corresponding transcriptome ROIs. The ratio $c^{\left(i\right)}/n^{\left(i\right)}$ associated with each feature is guaranteed to be a value between 0 and 1. To assess whether the observed overlap is due to chance, a random region is generated for each feature from the corresponding permutation space. The overlapping count between the randomized region set (without IA) and the ROIs is also evaluated. Each random region contributes either 0 or 1 to the null evaluation, which has the same range as the observed count $c^{\left(i\right)}/n^{\left(i\right)}$. In this way, observed evaluation and random evaluation can be compared based on the same scale, and meanwhile the isoform information is fully utilized.

Besides, RgnTX supports another measure of colocalization called coverage, which refers to the total number of overlapping nucleotides. The corresponding weighted measure is stated as follows:

$$W_{\mathrm{weigh}}:=\sum_{i=1}^{S}\left(\sum_{j=1}^{n^{\left(i\right)}}w_{j}^{\left(i\right)}/n^{\left(i\right)}\right)$$

where for the *i* -th feature, $n^{\left(i\right)}$ represents the count of its related isoforms, and $w_{j}^{\left(i\right)}$ represents the number of overlapping nucleotides between the feature and the ROIs on the *j*-th isoform. It calculates the sum of the average coverage of each feature with the ROIs as the observed measure.

Fig. 2**b** depicts a sketch map for the evaluation strategies. The observed evaluation is calculated by comparing features with ROIs. A null evaluation is generated by comparing randomized regions of features with the same ROIs. As permutation times increase, such null evaluations accumulate and form a null distribution. This distribution can be further analyzed to determine if the observed overlap is due to chance. Although the figure shows four different situations for evaluation in various cases, in the real application, users only need to choose a core permutation test function (either with or without IA) and specify the comparison metrics via corresponding parameters. All these can be done within only a few lines of code. Additionally, it is possible to define custom evaluation strategies as a function and pass them into the main function. The custom function should accept two sets of regions in the GRanges format and return a numeric value.

### 2.3 Summary statistics and visualization

The colocalization analysis of transcriptome elements can be easily performed by the core permutation test function within only a few lines of code. Most of the functions in RgnTX include an ellipsis operator, allowing the direct passing of arguments from auxiliary functions (such as randomization and evaluation functions) to the core permutation test function, making it simple to modify. To measure the statistical significance of the association being tested, a p-value and z-score are calculated. The p-value can be calculated based on either counting the percent of more extreme cases in random permutations than the real observation (default), or based on a t-test. The p-value can be chosen to be calculated based on either a one-tailed test or a two-tailed test. The z-score represents how many standard deviations an observed value is away from the mean of random evaluations, which serves as a numerical measurement describing the strength of the association being tested.

The core permutation testing and plotting functions are separated, allowing users to reproduce results by using intermediate outputs. The output of the core permutation testing function is summarized as an object, which includes randomized region sets, p-value, z-score and information about any detailed settings of the test. The plotting function takes this output and returns a ggplot2 object. Fig. 2**c** shows an example. A histogram depicts random evaluation results. Vertical lines in different colors indicate the observed evaluation (red) and the mean of random evaluations (green). A critical region is visualized to give an intuition of whether an association being tested is statistically significant or not. Fig. S2 provided an example of association between m^7^G sites and the 5′UTR, as well as the association between m^7^G sites and the CDS [34].

### 2.4 Multiple tests

RgnTX supports conducting multiple hypothesis testing with Benjamini-Hochberg correction. It reports the proportion of hypothesis tests with the null hypothesis being rejected among all tests based on a user-specified significance threshold. The Benjamini-Hochberg method is a widely used approach for multiple testing, which guarantees false discovery rate (FDR) is less than the significance level α for a series of *m* independent tests [35]. Although procedures based on family-wise error rate (FWER) control that directly control the probability of making at least one Type I error, such as Bonferroni and Holm-Bonferroni correction, provide a more stringent correction, they can be too strict for any discovery to be reported. The tests with FDR controlled, which control the expected proportion of false discoveries among all discoveries, have higher power than the tests with FWER-controlled, and are more suitable for this research.

The procedure of the Benjamini-Hochberg method is as follows:

- Sort the *m* p-values in increasing order.
- Denote the rank of each p-value *i*: $p^{\left(1\right)}\leq p^{\left(2\right)}\leq \ldots \leq p^{\left(i\right)}\leq \ldots \leq p^{\left(m\right)}$.
- Calculate candidate corrected p-values as:

$$p_{\mathrm{FDR}}^{\left(i\right)}=p^{\left(i\right)}\frac{m}{i}$$

- Starting from the (*m*-1)-th to the first item, apply the following instruction:

$$p_{\mathrm{FDR}}^{\left(i\right)}=\min(p_{\mathrm{FDR}}^{\left(i\right)},p_{\mathrm{FDR}}^{\left(i+1\right)})$$

Both p-values and Benjamini-Hochberg corrected p-values will be reported, also with the proportion of hypothesis tests being rejected among all tests based on a user-specified significance threshold α according to the following criterion:

- Find the maximum k such that
  $$p^{\left(k\right)}\leq \frac{k}{m}\alpha$$
- Reject all of $H_{0}^{\left(1\right)},H_{0}^{\left(2\right)},\ldots ,H_{0}^{\left(k\right)}$.

### 2.5 Shifted z-scores over transcripts

RgnTX supports the testing of z-scores with positional shifts to determine if an association is specifically tied to the exact position of regions. This involves shifting the original regions from their positions, reassessing the evaluation metric, and calculating new z-scores. The functions for calculating and plotting shifted z-scores in RgnTX follow a consistent protocol design. Calculating different shifted z-scores when the position of regions of interest is changed is supported by passing the output of the core permutation test function to the *shiftedZScoreTx* function. To be noticed, different from the *localZScore* function in regioneR, *shiftZScoreTX* in RgnTX can shift the transcriptome ROIs on mRNA rather than just on the genome coordinates.

Visualizing shifted z-scores is supported by passing the output of *shiftedZScoreTx* to the plotting function *plotShiftedZScoreTx*. If a peak is observed at the original point in the plot of shifted z-scores, it indicates a close relationship between the association and the specific position of corresponding transcriptome ROIs. We can see a clear peak in the center of the plot in Fig. 2**c**, suggesting the tested association is strongly affected by the exact positions of regions of interest. This figure also shows the scenario where the regions of interest are moved within a smaller window. To examine how such a smaller position shift affects the z-scores, users can assign smaller values to the window and step arguments in *shiftedZScoreTx*.

## 3 Results

### 3.1 RgnTX revealed distinct null distribution on the genome and transcriptome

In this section, we revealed that the null distribution of random overlaps on the genome and the transcriptome could be significantly different. We compared random overlaps from the following genome- or transcriptome-related backgrounds. All the backgrounds were region sets related to the same (randomly picked) 300 genes in hg19. We used the annotation database from UCSC to extract the following backgrounds: the 'DNA' background, which consists of the region set of the 300 genes; the 'exonic DNA' background, which includes a portion of the 300 genes that will form mature RNA; the 'pre-mRNA' background, which includes all the full transcripts related to the 300 genes; and the 'mRNA' background, which includes all the mature RNAs related to the 300 genes. After preparing these four backgrounds, simulated data were generated from each background type. In each background, 1000 pairs of region sets were randomly picked to compare the overlap of each pair, with each region set containing 500 regions of the same length.

**Fig. 3:**
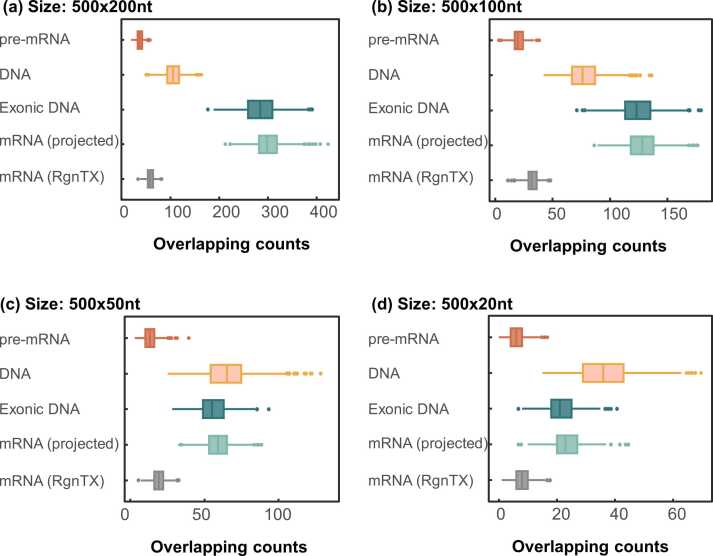
Null distributions of random overlaps over the genome and the transcriptome are significantly different. In each subplot, 1000 pairs of random region sets from the four different spaces were generated, with each region set containing 500 regions of the same length. The number of overlaps between each pair was counted and visualized. Only a small number of overlaps were observed on the transcriptome (the last group in each subplot), which are significantly fewer than the results from the DNA, exonic DNA and mRNA (projected to the genome) spaces, and generally larger than that on the pre-mRNA space. Box boundaries represent the 25th and 75th percentiles; center line represents the median. These results suggested that the permutation of heterogeneous transcriptome elements could be substantially more complex than genome-based elements.

Although all the backgrounds were restricted to only 300 genes, the overlapping counts in each background were significantly different. The evaluation results were shown in Fig. 3. The random overlaps on the ‘DNA’, ‘exonic DNA’ and ‘pre-mRNA’ background were counted at the genome level using the regioneR function *numOverlaps* [23]. On the other hand, the overlaps in ‘mRNA’ background were not only evaluated at the genome level (projected to the genome), but also counted at the transcriptome level by the RgnTX function *overlapCountsTx*. It is not surprising that only a small number of overlaps were observed on the transcriptome by RgnTX due to the existence of multiple isoform transcripts. Interestingly, the results from the ‘exonic DNA’ and the ‘mRNA (projected)’ also differed, which could be attributed to the varying frequencies at which exons are utilized by isoform transcripts. Additionally, a two-sample t-test was conducted to evaluate the difference in the null distribution of random overlaps on ‘mRNA (projected)’ and overlaps on other backgrounds (See Table S1).

These results suggest that the permutation of heterogeneous transcriptome elements is considerably more complex than that of genome-based elements. Therefore, the conventional genome-based methods are not capable of characterizing the randomness of the transcriptome and analyzing transcriptome elements. This highlights the need for the development of RgnTX, a colocalization analysis tool that can conduct permutation test at the transcriptome level.

### 3.2 Case study: colocalization analysis of association between m^6^A and stop codons

To demonstrate the usage of RgnTX, we conducted a study to test the association between m^6^A RNA methylation sites (with isoform ambiguity) from miCLIP-seq [36] and stop codon regions (without isoform ambiguity) derived from UCSC transcriptome annotation.

In this case study, we compared four testing modes, which differed in two aspects Firstly, whether the permutation background included all transcripts or only the m6A-contained transcripts. Secondly, whether the permutation test was conducted at the genome level or at the transcriptome level. These different combinations resulted in four testing modes. It is important to note that the modes conducted at the genome level ignored transcriptome heterogeneity and isoform information, while the other two modes at the transcriptome level preserved isoform information using the isoform-aware permutation strategy proposed by the RgnTX package.

**Fig. 4:**
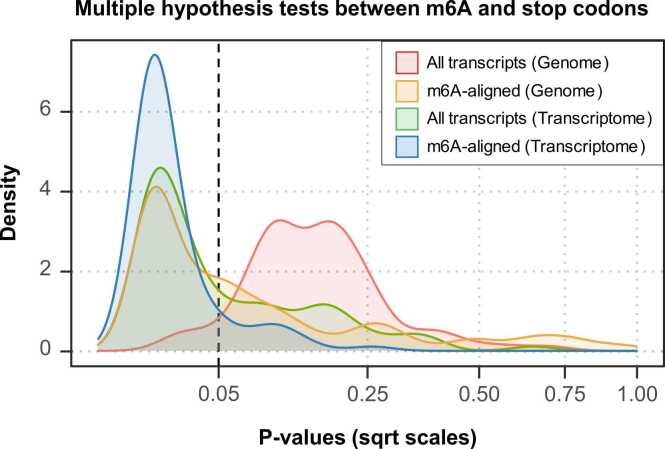
Density plot of p-values of multiple tests assessing association between m^6^A RNA methylation sites and stop codon regions based on different permutation strategies and backgrounds. Conventional genome-based permutation test strategies and RgnTX-provided isoform-aware permutation strategies were performed with the same m^6^A datasets derived from the miCLIP-seq technology and the same stop codons provided by USCS hg19. The novel isoform-aware strategies (Transcriptome) provided by RgnTX returned more significant signals compared to the conventional genome-based strategies (Genome).

For each of the four testing modes, we performed multiple hypothesis tests as follows. We randomly shuffled all the m^6^A sites and divided them into independent groups. Each independent group contains 100 regions and does not contain repeated regions with each other. Next, we conducted permutation test for each group, where association between m^6^A sites and the stop codon regions were evaluated with 100 permutation times. Each group returned a p-value calculated by counting the percent of extreme values in random permutations than the real observation. We visualized the p-values for multiple groups in Fig. 4. The transcriptome-based m^6^A-aligned permutation mode showed the most significant results, with a p-value < 0.05 in 89.01 % of groups, while the conventional genome-based approach involving all transcripts only reported 5.49 % of significant cases. More detailed results were shown in Table 1. Even with different significance threshold settings, the transcriptome-based m^6^A-aligned permutation mode consistently reported the largest percentage of multiple tests rejecting the null hypothesis. It continued to detect the most significant signals as the sample size and permutation time increased to 500 for each group (see Table S2).

**Table 1:** p-values of multiple tests between m^6^A sites and stop codons.

p-values	All transcripts (Genome)	m^6^A-aligned (Genome)	All transcripts (Transcriptome)	m^6^A-aligned (Transcriptome)
Mean	0.1767	0.1321	0.0786	0.0254
Median	0.1683	0.0396	0.0198	0.0099
< 0.05 (%)	5.494	58.24	63.73	89.01
< 0.1 (%)	21.98	71.43	74.73	93.41
< 0.2 (%)	70.32	80.22	89.01	98.90

Note: Mean, median of p-values and percent of p-values less than different significance levels. RgnTX permutation test strategies (Transcriptome) returned more signals compared to the conventional strategies (Genome) no matter the background involves all the transcripts or only the m^6^A-contained transcripts.

Moreover, RgnTX provided Benjamini-Hochberg correction to adjust the p-values, reducing the number of false positives. Taking the same case shown in Table 1 as an example, after correction, the transcriptome-based permutation modes still reported a high percent of significant results (see Table 2), while interestingly, the conventional genome-based mode conducted on all transcripts failed to report any multiple tests that rejecting the null hypothesis.

**Table 2:** Adjusted p-values of multiple tests between m^6^A sites and stop codons.

p-values	All transcripts (Genome)	m6A-aligned (Genome)	All transcripts (Transcriptome)	m6A-aligned (Transcriptome)
Mean	0.2965	0.1613	0.1010	0.0299
Median	0.2761	0.0751	0.0392	0.0143
< 0.05 (%)	< 1.09	43.96	50.55	87.91
< 0.1 (%)	< 1.09	62.64	65.93	92.31
< 0.2 (%)	< 1.09	78.02	82.42	98.90

Note: Similar to Table 1, except that the p-values were adjusted by the Benjamini-Hochberg correction, which excluded potential false positives. After correction, the isoform-aware methods still reported significant signals, while the genome-based permutation test strategy that involved all transcripts (the first column) failed to report any significant results.

To examine the robustness of conclusions, we compared RgnTX with other methods being run with its default mode [8], [12], [13], [16], [17], [27]. We performed multiple hypothesis tests with the same m^6^A datasets as input and summarized the results in Table S3. We categorized these methods according to the test statistics they relied on. The results indicated consistency in the conclusions across different analytical models. We also examined influence of the chromosomal location of m^6^A on the outcomes of multiple hypothesis testing (See Fig. S3). The results of these tests, conducted on individual chromosomes, were found to be generally consistent with the overall findings obtained from analyzing the entire transcriptome.

## 4 Discussion

Recently, there has been significant progress in the method development for colocalization analysis of genomic elements. This analysis is crucial for understanding potential functional interactions between corresponding biological attributes and provides hints for further investigation. The null hypothesis model should be selected carefully, since a slight deviation from the real scenario of the background may lead to biased results. Unfortunately, existing methods do not explicitly consider transcriptome heterogeneity and isoform ambiguity, making them less suitable for analyzing transcriptome elements. Current methods are usually based on the assumption that genomic features occur independently along the genome, and they generate a null model by shuffling region sets across the entire genome or chromosomes. They fail to model a suitable null transcriptome background or generate transcriptome randomized region sets. Synthetic data examples have shown that the null distributions of random overlaps over the genome and the transcriptome can be significantly different, suggesting the necessity of transcriptome-specific colocalization method.

Motivated by these limitations, we developed RgnTX, a tool designed to perform colocalization analysis at the transcriptome level. Similar to regioneR and other permutation methods at the genome level, RgnTX incorporates randomization, evaluation, and z-score shifting. However, RgnTX differs from regioneR in several important aspects. Firstly, RgnTX provides randomization functions that can shuffle regions within a transcriptome background. It allows for the direct sampling of regions over mRNA space. Although regioneR provides variables for users to mask the regions that they do not want to work with, it cannot be applied to pick random regions across alternative splicing junctions, i.e., picking a consecutive region that spans more than one exon.

Secondly, RgnTX supports transcript-specific permutation, whereas regioneR is primarily designed for permutation over chromosomes. In the evaluation section, RgnTX distinguishes evaluation strategies that count overlaps at the genome and transcriptome level. Importantly, RgnTX introduces an innovative isoform-aware permutation mode to address the isoform ambiguity problem in colocalization analysis for the first time. This mode counts each potential overlap on all isoforms and calculates an average. By doing so, RgnTX ensures that observed evaluation and random evaluation can be compared on the same scale, while fully utilizing isoform information. Thirdly, in the z-score shift section, RgnTX introduces the *shiftZScoreTX* function, which supports the shifting of regions of interest on corresponding mRNA regions rather than solely on genome coordinates. Calculation and plotting functions related to shifted z-scores are separated and follow a consistent protocol design, allowing for more convenient analysis and interpretation. All these innovations make RgnTX a reliable tool for conducting colocalization analysis at the transcriptome level.

## 5 Conclusions

RgnTX served as a tool for the colocalization analysis of transcriptome elements with isoform-aware permutation tests. Different from conventional genome-based approaches, RgnTX enables the shuffling with user-decided restriction of areas at transcriptome level and offers high flexibility in the null model to simulate the realistic transcriptome-wide background. An important contribution here is the support of the testing of transcriptome elements without clear isoform association, which is often the real scenario due to technical limitations. The case study (m^6^A RNA methylation data and stop codons) showed that colocalization analysis of genomic features with isoform ambiguity problem could be stably addressed by our novel strategies. A consistent protocol design is implemented for the most of functions to make the package easy to use. Users are able to conduct colocalization analysis about tailored experiments within only a few lines of codes. To sum, RgnTX designs novel permutation strategies that preserve the characteristics of transcriptome elements, effectively tackles the isoform ambiguity problem, easy to be utilized by researchers to generate any null models in the context of transcriptome and provides various statistical calculation and visualization functions for conducting colocalization analysis.

## Code availability

The package is freely available at: https://github.com/yue-wang-biomath/RgnTX. It has been accepted by Bioconductor, and the package landing page is at: https://bioconductor.org/packages/RgnTX.

## Funding

The work was supported by the 10.13039/501100001809National Natural Science Foundation of China [32100519 and 31671373] and the XJTLU Key Program Special Fund [KSF-E-51 and KSF-P-02].

## CRediT authorship contribution statement

**Yue Wang:** Conceptualization, Methodology, Software, Investigation, Formal analysis, Writing – original draft. **Zhen Wei:** Data curation, Writing – original draft. **Frans Coenen:** Resources, Supervision. **Jionglong Su:** Resources, Supervision. **Jia Meng:** Resources, Supervision.

## Declaration of Competing Interest

All authors declare that they have no known competing financial interests or personal relationships that could have appeared to influence the work reported in this paper.
