# Supplementary material for: RgnTX: Colocalization analysis of transcriptome elements in the presence of isoform heterogeneity and ambiguity
Source: Comput Struct Biotechnol J. 2023 Aug 24;21:4110–7. doi: 10.1016/j.csbj.2023.08.021 (PMC10475473; doi:10.1016/j.csbj.2023.08.021)
Supplement: Supplementary file 1 — Supplementary material [file mmc1.docx]

Supplementary Materials

**RgnTX: colocalization analysis of transcriptome elements in the presence of isoform heterogeneity and ambiguity**

Yue Wang^1,5^, Zhen Wei^2,6^, Jionglong Su^3^, Frans Coenen^5^ and Jia Meng^2,4,6*^

^1^Department of Mathematical Sciences, ^2^Department of Biological Sciences, ^3^School of AI and Advanced Computing, ^4^AI University Research Centre, Xi’an Jiaotong-Liverpool University, Suzhou, Jiangsu, 215123, China; ^5^Department of Computer Science, ^6^Institute of Systems, Molecular and Integrative Biology, University of Liverpool, L69 7ZB, Liverpool, United Kingdom.

^*^To whom correspondence should be addressed: [jia.meng@xjtlu.edu.cn](mailto:jia.meng@xjtlu.edu.cn) (JM).

**Table S1.** p-values of association between random overlaps on the mRNA (RgnTX) background and overlaps on other four backgrounds.

| **Sizes** | **pre-mRNA** | **DNA** | **exonic DNA** | **mRNA (projected)** |
| --- | --- | --- | --- | --- |
| 500 X 200nt | 5.2808e-53 | 1.7689e-67 | 9.0495e-89 | 1.3848e-103 |
| 500 X 100nt | 9.7254e-45 | 4.9964e-52 | 1.0446e-74 | 2.2628e-88 |
| 500 X 50nt | 2.7784e-10 | 6.8063e-53 | 5.1256e-61 | 1.1597e-74 |
| 500 X 20nt | 5.6318e-05 | 5.3277e-66 | 4.2660e-57 | 3.9651e-57 |

Note: A two-sample t-test was conducted to evaluate the difference in the null distribution of random overlaps on heterogenous transcriptome and overlaps on other backgrounds.

**Table S2.** p-values of multiple tests between m^6^A sites and stop codons with each group involving 500 regions and 500 permutation times.

| **p-values** | **All transcripts**  **(Genome)** | **m^6^A-aligned**  **(Genome)** | **All transcripts**  **(Transcriptome)** | **m^6^A-aligned**  **(Transcriptome)** |
| --- | --- | --- | --- | --- |
| Mean | 0.1321 | 0.0569 | 0.0057 | 0.0022 |
| Median | 0.1238 | 0.0019 | 0.0019 | 0.0019 |
| < 0.01 (%) | < 5.26 | 73.68 | 94.74 | 100 |
| < 0.005 (%) | < 5.26 | 78.95 | 89.47 | 94.74 |

Note: Mean, median of p-values and percent of p-values less than different significance levels. Compared to Table 1, p-values were smaller due to the increase of sample sizes and permutation times. The transcriptome-based m^6^A-aligned permutation mode still returned the most significant results.

**Table S3.** Multiple hypothesis tests between m^6^A sites and stop codons based on different test statistics.

| **Methods** | **Statistics** | **p-values** | **Not consider IA** | | **Consider IA** | |
| --- | --- | --- | --- | --- | --- | --- |
|  |  |  | **All transcripts**  **(Genome)** | **m^6^A-aligned**  **(Genome)** | **All transcripts**  **(Transcript)** | **m^6^A-aligned**  **(Transcript)** |
| RgnTX | t-test | Mean | 2.4e-02 | 2.2e-02 | 7.8e-03 | 4.6e-06 |
|  |  | Median | 6.9e-16 | 5.2e-41 | 1.4e-42 | 2.9e-60 |
| RgnTX (default)  MULTOVL  HyperBrowser | extreme obs  /random num | Mean | 0.1767 | 0.1321 | 0.0786 | 0.0254 |
|  |  | Median | 0.1683 | 0.0396 | 0.0198 | 0.0099 |
| GIGGLE  LOLA | fisher exact  test | Mean | 0.2407 | 0.1203 | 0.0574 | 0.0492 |
|  |  | Median | 0.1640 | 0.1121 | 0.0327 | 0.0317 |
| Genome  -Runner | chi-square  test | Mean | 0.2396 | 0.1224 | 0.0592 | 0.0513 |
|  |  | Median | 0.1658 | 0.1137 | 0.0339 | 0.0339 |

Note: We performed multiple hypothesis with the same m^6^A datasets as input. To be noticed, all the other methods [1-6] we cited are only able to perform colocalization tests without considering IA (the first and the second columns). The cases considering IA shown in the last two columns were facilitated by RgnTX randomization functions and the p-values were calculated according to corresponding test statistics.

**Reference**

1. Sandve G. K., Gundersen S., Rydbeck H., et al., *The Genomic HyperBrowser: inferential genomics at the sequence level.* Genome Biology, 2010. **11**(12): p. R121.

2. Aszódi A., *MULTOVL: fast multiple overlaps of genomic regions.* Bioinformatics, 2012. **28**(24): p. 3318-3319.

3. Sheffield N. C. and Bock C., *LOLA: enrichment analysis for genomic region sets and regulatory elements in R and Bioconductor.* Bioinformatics, 2016. **32**(4): p. 587-589.

4. Dozmorov M. G., Cara L. R., Giles C. B., et al., *GenomeRunner web server: regulatory similarity and differences define the functional impact of SNP sets.* Bioinformatics, 2016. **32**(15): p. 2256-2263.

5. Layer R. M., Pedersen B. S., DiSera T., et al., *GIGGLE: a search engine for large-scale integrated genome analysis.* Nature Methods, 2018. **15**(2): p. 123-126.

6. Simovski B., Kanduri C., Gundersen S., et al., *Coloc-stats: a unified web interface to perform colocalization analysis of genomic features.* Nucleic Acids Research, 2018. **46**(W1): p. W186-W193.

**
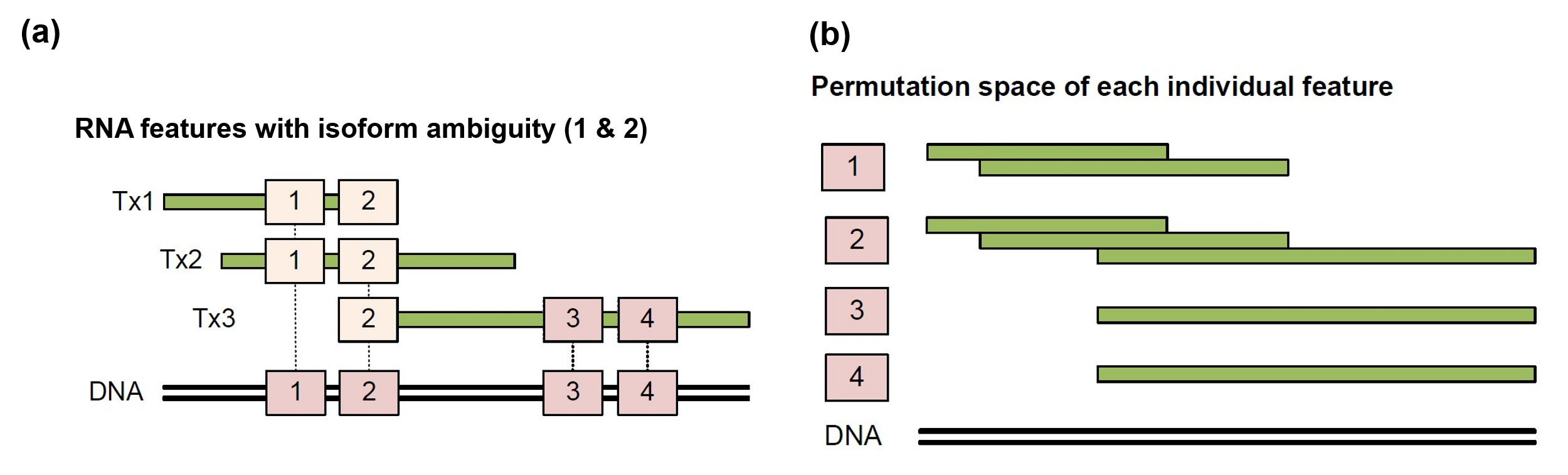
**

**Figure S1.** The default randomization strategy in RgnTX. (a) RNA features to be randomized. Feature 1 and 2 have isoform ambiguity, which can be aligned to multiple isoforms. Feature 1 and 2 may come from any of the transcripts they align with. (b) The default randomization space for each individual RNA feature. The randomized regions will be generated from the same transcript (or potential transcripts) as the original RNA features


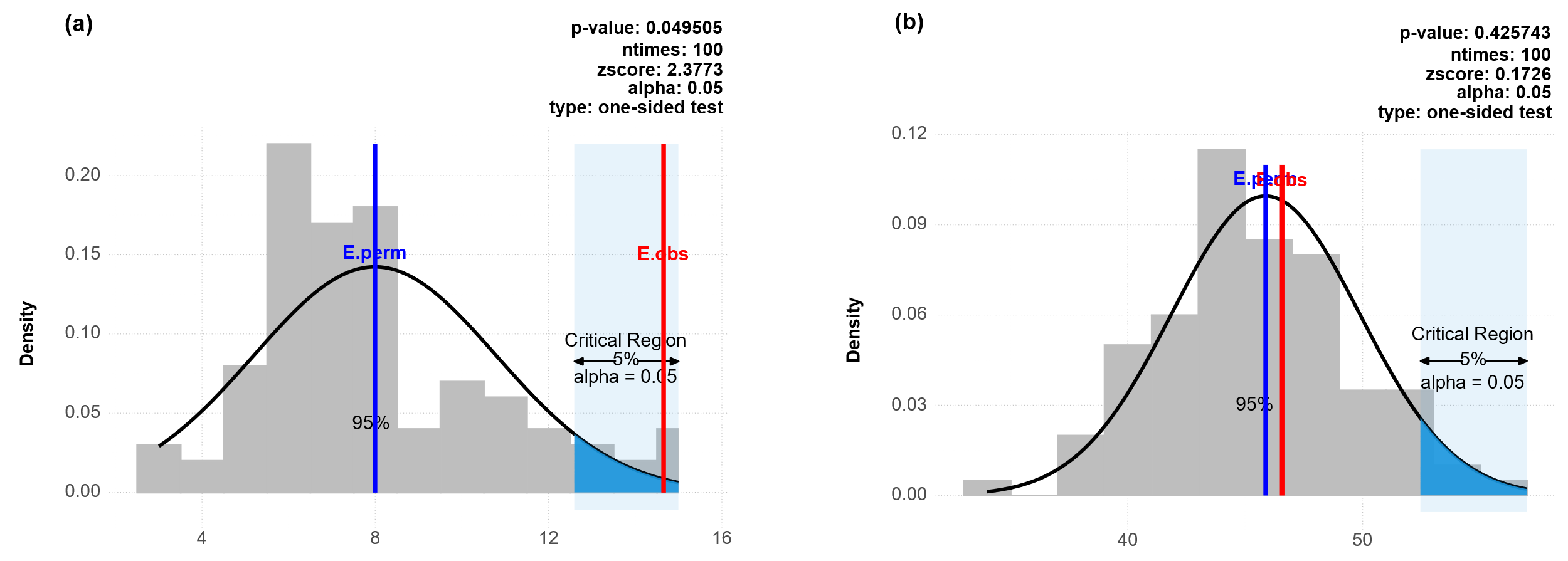


**Figure S2.** A case study examining (a) the association between m^7^G sites and the 5' untranslated region (UTR), as well as (b) the association between m^7^G sites and the coding sequence (CDS).


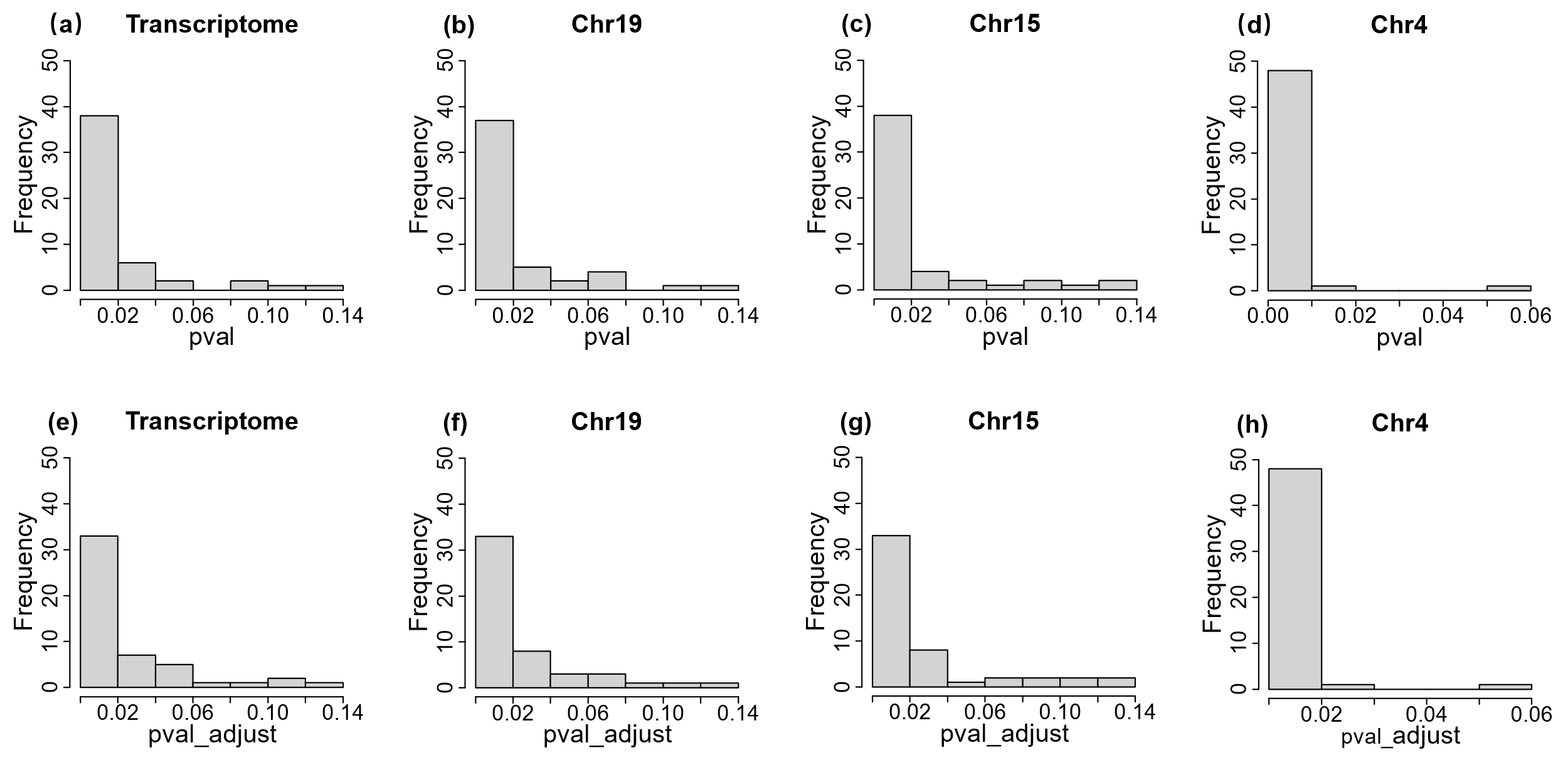


**Figure S3.** Multiple hypothesis tests between the association between m^6^A sites and stop codons on the whole transcriptome and on different chromosomes. (a), (b), (c) and (d) visualize the p-values of each case. (e), (f), (g) and (h) demonstrate adjusted p-values.
